# Effectiveness of Apigenin, Resveratrol, and Curcumin as Adjuvant Nutraceuticals for Calvarial Bone Defect Healing: An In Vitro and Histological Study on Rats

**DOI:** 10.3390/nu15051235

**Published:** 2023-02-28

**Authors:** Felice Lorusso, Antonio Scarano, Stefania Fulle, Luca Valbonetti, Rosa Mancinelli, Ester Sara Di Filippo

**Affiliations:** 1 Department of Innovative Technologies in Medicine & Dentistry, University of Chieti-Pescara, Via dei Vestini 31, 66100 Chieti, Italy; 2 Department of Neuroscience, Imaging and Clinical Sciences, University “G. d’Annunzio” of Chieti-Pescara, 66100 Chieti, Italy; 3 Faculty of Bioscience and Agro-Food and Environmental Technology, University of Teramo, 64100 Teramo, Italy

**Keywords:** nutraceuticals, bone healing, polyphenols, flavonoids, adjuvant supplement

## Abstract

Bone healing is a major clinical issue, especially in bone defects of critical dimensions. Some studies have reported in vivo positive effects on bone healing by some bioactive compounds, such as the phenolic derivatives found in vegetables and plants, such as resveratrol, curcumin, and apigenin. The aim of this work was (1) to analyze in vitro in human dental pulp stem cells the effects of these three natural compounds on the gene expression of related genes downstream to *RUNX2* and *SMAD5*, key factor transcriptions associated with osteoblast differentiation, in order to better understand the positive effects that can occur in vivo in bone healing, and (2) to evaluate in vivo the effects on bone healing of critical-size defects in the calvaria in rats of these three nutraceuticals tested in parallel and for the first time administered by the gastric route. Upregulation of the *RUNX2*, *SMAD5*, *COLL1*, *COLL4*, and *COLL5* genes in the presence of apigenin, curcumin, and resveratrol was detected. In vivo, apigenin induced more consistent significant bone healing in critical-size defects in rat calvaria compared to the other study groups. The study findings encourage a possible therapeutic supplementation with nutraceuticals during the bone regeneration process.

## 1. Introduction

The treatment of critical-size bone defects in humans, severe maxillary atrophies and long-bone critical-size defects, often requires a multidisciplinary approach and extensive bone grafting [1,2]. The adoption of the appropriate animal study design gives reliable data and translational application to human bone defects [3,4]. The critical-size bone defect represents an orthotopic model where the hard tissue is not able to heal spontaneously with no intervention [5]. Experimental calvaria critical-size defects are histologically characterized by focal competition between inflammatory tissues and new bone formation [5]. This model has been validated for the evaluation of the biological effects of biomaterials to bridge nonunion defects. In addition, it is optimal to investigate the effects of adjuvant supplements on osteogenesis and bone maturation [6]. Human dental pulp stem cells (hDPSCs) are mesenchymal stem cells (MSCs) capable of both self-renewal and differentiation according to an osteogenic phenotype. In recent years, this capability has been proposed for tissue engineering and cell seeding on biomaterial to ameliorate new bone formation and graft osseointegration [7]. In addition, the osteogenic differentiation properties of MSCs are determinants to sustain the regenerative process [8]. Tissue engineering aims at the structural and functional restoration of damaged tissues through MSC differentiation protocols alone or complexed with biological scaffolds to produce a tissue neoformation immunologically, functionally, structurally, and mechanically identical to the native one [3]. Nutraceuticals represent bioactive compounds, products derived from food sources, characterized by medical or healthy benefits, including prevention and protection against several systemic diseases [9,10,11,12]. Indeed, bioactive compounds are involved in many physiological and pathophysiological processes as tissue damage repair or protection from chronic diseases and cellular oxidative stress [10,12,13,14]. Some in vivo studies have reported positive effects on bone healing by some bioactive compounds, such as the phenolic derivatives found in vegetables and plants, such as resveratrol, curcumin, and apigenin [11,12,13,14,15]. As reported by several studies, resveratrol is a polyphenol with antioxidant, anti-inflammatory, and antiaging properties [15]. In the literature, resveratrol has been evaluated in association with three-dimensional-cell–engineered scaffolds, showing the promotion of osteogenesis and the overexpression of the runt-related transcription factor 2 (*RUNX2*) and osteocalcin (*OCN*) genes [16,17,18]. In mice, resveratrol administered in combination with insulin produced a significant increase in new bone formation of critical-size defects in the calvaria in animals affected by diabetes. Furthermore, the combination of insulin and resveratrol induced the modulation of bone morphogenetic protein type 2 (*BMP-2*) gene expression [19,20]. Apigenin is a flavonoid commonly found in different plants (such as chamomile) and vegetables, and it is recognized for its antioxidant, anti-inflammatory, and protective properties in chronic diseases [21,22,23]. However, very little information is available about its effects on bone metabolism [24,25,26]. Zhang et al. reported that apigenin promotes osteogenic differentiation in MSCs via the *JNK* and *p38 MAPK* pathways, through increased expression of *RUNX2* and osterix (*OSX*) proteins [23]. Furthermore, other studies have reported that apigenin inhibits osteoclastogenesis and osteoclast function [25,27,28]. Curcumin is a natural polyphenolic phytochemical that is characterized by a total of seven carbon linkers with three major functional groups, including α,β-unsaturated β-diketone with an aromatic O-methoxy-phenolic functional group [29,30,31]. Curcumin is able to modulate cytokines, growth factors, transcription factors, and inflammatory molecules through different pathways [32,33,34]. The principal way is associated with the inhibition of the transcription by nuclear factor-kappa B (*NF-kB*) [15]. As described in the literature, this molecule has a protective and preventive effect against oral cancer and several metabolic diseases [15]. In tissue engineering, it has been reported that curcumin elution nanopolymers produce in vitro an increased gene and protein expression of osteogenic markers *RUNX2*, *ALP*, and *BMP2* [15,35,36]. Altogether, the cited studies have demonstrated that all three compounds induced an increase in gene and/or protein expression of *RUNX2*. To the best of our knowledge, however, no studies have been carried out on a simultaneous analysis of these three compounds and which pathways downstream of *RUNX2* are modulated in the process of osteogenesis both in vivo and in vitro. Furthermore, even if these compounds exhibit a protective function of bone physiology [37], their role in bone fracture should be clarified [38]. The only study involving these three phenolic compounds concerns their effects in inducing cancer signaling pathway manipulation and possibly facilitating new treatment modalities for osteosarcoma [39]. Therefore, the aim of the present study was to (1) analyze in vitro in hDPSCs, in the presence of these three natural compounds, the gene expression downstream of *RUNX2* and *SMAD5*, key factor transcriptions associated with osteoblast differentiation, in order to better understand the positive effects that can occur in vivo in bone healing; and (2) evaluate in vivo the effects of these three nutraceuticals used in parallel on the bone healing of critical-size defects in rat calvaria using an innovative method of administration by gastric gavage in a single dose daily repeated for 30 consecutive days. To this purpose, in vitro experiments were performed on hDPSCs in the presence of these three natural compounds added individually, and the gene expression of *RUNX2*, *SMAD5*, *COLL1*, *COLL4*, and *COLL5* was analyzed. These substances can prove to be effective in the field of regenerative medicine as they modulate molecular mechanisms, which can therefore increase osteogenic differentiation and consequently improve bone regeneration. The investigated molecules could be considered promising supplements able to repair bone defect healing. The null hypothesis considered no differences in osteogenic properties and bone repair capability between the control and the different nutraceuticals groups.

## 2. Materials and Methods

### 2.1. Dental Pulp Stem Cells Cultures

Human dental pulp stem cells (hDPSCs) were obtained by stem cell banks (#PT-5025, Lonza, Walkersville, MD, USA) and maintained at −80 °C. The hDPSCs were defrosted and cultured with a growth medium (GM) obtained by DPSC basal medium (#PT3927, Lonza, Walkersville, MD, USA), supplemented with the DPSC SingleQuots™ Kit (#PT4516, Lonza, Walkersville, MD, USA) and incubated at 37 °C and 5% CO_2_. The GM was changed twice a week, just before cells became confluent (subconfluent). Once the cells reached an adequate number, they were washed twice with phosphate-buffered saline (PBS) (#ECB4004L, Euroclone, Milan, Italy) and detached using 1 mL Trypsin–EDTA 1× in PBS (#ECB3052D, Euroclone, Milan, Italy) for 5 min at 37 °C. The cells were collected in a sterile tube and centrifuged for 5 min at 900 rpm. Once resuspended, the cells were counted by a Bürker chamber and used for further experiments. The differentiation medium (DM) was obtained by supplementation of Human Mesenchymal Stem Cell Osteogenic Differentiation Basal Medium (#PT-3924, Lonza, Walkersville, MD, USA) with hMSC Osteogenic SingleQuots (#PT-4120, Lonza, Walkersville, MD, USA) and used to evaluate osteogenic differentiation.

### 2.2. In Vitro Study Design

Human DPSCs were cultured as described above in GM for 24 and 72 h to perform the proliferation rate assay and in DM for 14 days to perform Alizarin Red and gene expression analysis in four experimental conditions: hDPSCs (control), hDPSCs + apigenin (#10798, Sigma-Aldrich, Saint Louis, MO, USA), hDPSCs + resveratrol (#R5010, Sigma-Aldrich, Saint Louis, MO, USA), hDPSCs + curcumin (#08511, Sigma-Aldrich, Saint Louis, MO, USA). The molecules were prepared in DMSO (#D5879, Sigma-Aldrich, Saint Louis, MO, USA) to avoid an immediate decomposition, according to the guidelines.

### 2.3. Cell Proliferation Assay

Cells were seeded in 96-well plates at a density of 1.6 × 10^3^ cells/well in 0.2 mL medium. After 2 h, the cells were stimulated with high or low concentrations of our compounds. High concentration: 1, 5, and 10 µM apigenin; 1, 10, and 50 µM resveratrol; and 1, 10, and 100 µM curcumin; low concentration: 100 and 500 nM and 1 µM apigenin; 100 and 500 nM and 1 µM resveratrol; 50, 100, and 500 nM curcumin. Cell proliferation in the presence of high concentrations was followed for 24 and 48 h and in the presence of low concentrations was followed for 24 and 72 h. At the end of each incubation interval, 3-(4,5-dimethylthiazol-2-yl)-2,5-diphenyl-tetrazolium bromide (MTT, #M5655 Sigma-Aldrich, Saint Louis, MO, USA) was added to each well to a final concentration of 0.5 mg/mL. The plates were incubated for 3 h at 37 °C and then centrifuged at 500× *g*. The supernatants were removed and discarded, and 200 µL dimethyl sulfoxide (DMSO, #D5879, Sigma-Aldrich, Saint Louis, MO, USA) was added. After incubating for 30 min at 37 °C, the absorbance was determined by spectrophotometry (SpectraMAX 190) at a wavelength of 560 nm.

### 2.4. Alizarin Red Staining

Cells were plated in 6-well plates at a density of about ~20,000 cells/well. After 24 h, GM was replaced with DM, and stimuli were added as follows: 1 µM apigenin, 100 nM resveratrol, and 50 nM curcumin. The Alizarin Red assay was performed to evaluate the mineralized nodule deposit in hDPSCs after 14 days. Cells were washed twice in PBS and then fixed with 1 mL/well of 4% paraformaldehyde (#157–8, Electron Microscopy Sciences, Hatfield, PA, USA) for 15 min. Specimens were washed twice with deionized water, and 1 mL/well of 1% Alizarin Red 40 nM (#A5533, Sigma-Aldrich, Saint Louis, MO, USA) was added and incubated for 20 min at room temperature. Specimens were then washed four times with deionized water for 5 min and viewed under a light microscope at a magnification of 10×.

### 2.5. Quantitative Real-Time PCR for Gene Expression Analysis

Cells were plated at a density of 2 × 10^3^ cells/cm^2^. After 24 h, GM was replaced with DM, and stimuli were added as follows: 1 µM apigenin, 100 nM resveratrol, and 50 nM curcumin. The cells were stimulated at these specific concentrations based on the results obtained with the cell proliferation assay. After 14 days of differentiation, hDPSCs were harvested for RNA extraction and real-time PCR analysis. The total RNA was isolated using Tri Reagent (#T9424, Sigma-Aldrich, Saint Louis, MO, USA), according to the manufacturer’s protocol. A quantity of 1 µg RNA was directly processed by High-Capacity cDNA Archive Kits (Applied Biosystems, Life Technologies, Monza-Italy) according to the manufacturer’s instructions. Singleplex real-time PCR was conducted to evaluate the relative quantification of gene expression of *RUNX2*, *SMAD5*, *COLL4*, *COLL5*, and *COLL1* versus *GAPDH* by TaqMan technology on an ABI Prism 9700HT Sequence Detection System instrument, connected to Sequence Detector Software (SDS, version 2.0; Applied Biosystem, Life Technologies, Monza, Italy) for data collection and analysis. The primer pairs and TaqMan probes for all of the target genes and for the *GAPDH* reference gene were provided as 20× mixtures that were ready to use at a concentration of 1×. According to the manufacturer’s recommendations, 25 µL reactions were performed in a MicroAmp Optical 96-well reaction plate using the 12.5 µL 2× TaqMan Universal PCR Master Mix, with the 1.25 µL 20× Inventoried Gene Expression Product for the mouse *Runx2* target gene, *SMAD5*, *COLL1*, *COLL4*, and *COLL5* versus *GAPDH* (FAM-dye-labeled TaqMan MGB probe). PCR was performed at 50 °C for 2 min, and at 95 °C for 10 min, and then run for 45 cycles at 95 °C for 15 s and at 60 °C for 1 min. All of the reactions were run in triplicate, and each experiment was repeated three times. The relative quantification of target gene expression was evaluated with data from the SDS software, using the arithmetic formula 2^−ΔΔCt^, according to the comparative Ct method, which represents the amount of target, as normalized to the *GAPDH* endogenous control. Data derived from the 2^−ΔΔCt^ formula are named relative quantification.

### 2.6. In Vivo Animal Study

#### 2.6.1. Surgical Procedure

**Figure 1:**
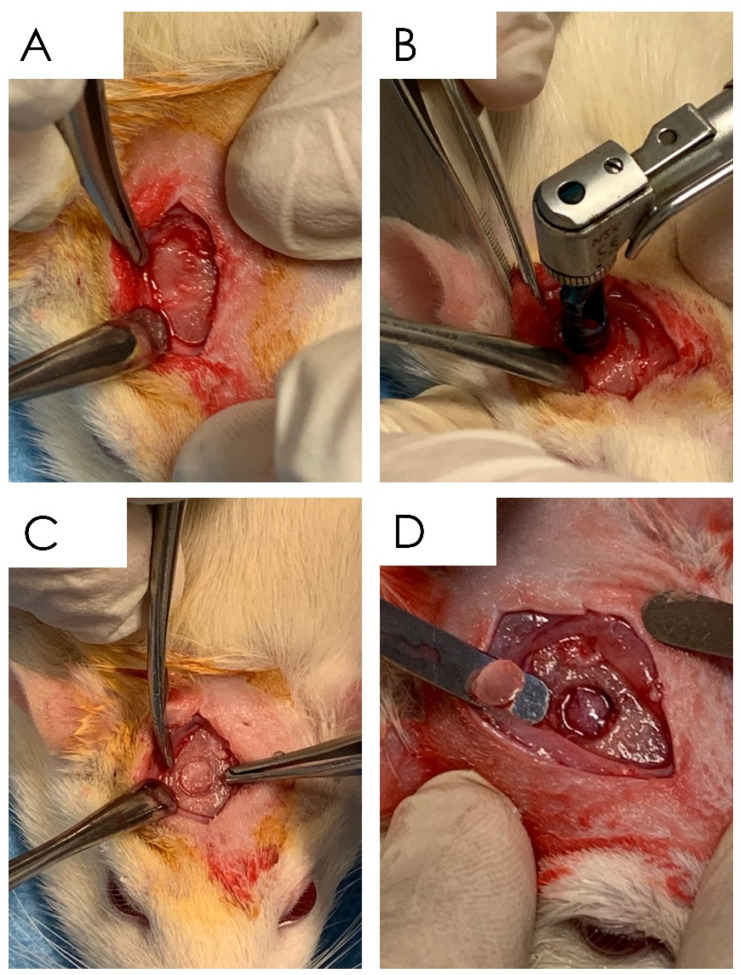
Surgical phases of the in vivo experiment: (**A**) exposure of the calvarial surface through a full-thickness incision; (**B**) drilling of the cortical surface of the calvaria through the 5 mm trephine bur; (**C**) details of the site after the osteotomy; (**D**) cortical layer removal.

The study received the approval of the ethical committee of the local Ethics Committee of the University of Chieti-Pescara, Chieti, Italy (No. 84/2020), and the Italian Ministry of Health. Twenty adult male Sprague Dawley rats were used for this study. Bone defects were produced in the calvaria bone (Figure 1A–D). Anesthesia was obtained by an intraperitoneal injection of sodium pentobarbital (Pentobarbital, Italy, 50 mg/kg). After shaving, the surgical field was prepared with 10% iodine solution. A sagittal incision of the midline was made starting from the occipital region and proceeding with the periosteal dissection highlighting the parietal region. The unilateral cranial bone defect (diameter, 5 mm) (1 defect/rat) was produced by using a drill under abundant irrigation of sterile physiological solution. After removing the bone disc, the various planes were sutured. Pain relieving and antibiotic therapy were administered with the methods and dosages previously described [5,20]. Twenty experimental defects were created (Figure 1A–D):

(1) Group A: Ctr—empty bone defect;
(2) Group B: Resveratrol (resveratrol 98%, No. 3183, Galeno SRL, Comeana, Italy) 1 mL (10 mg/kg)—empty bone defect/administration of resveratrol by gastric gavage in a single dose daily [40];
(3) Group C: Curcumin (curcumin 95%, No. 4507, Galeno SRL, Comeana, Italy) 1 mL (10 mg/kg)—empty bone defect/administration of curcumin by gastric gavage in a single dose daily [41];
(4) Group D: Apigenin (apigenin 98%, Biorigins, Sandleheath, UK) 1 mL (10 mg/kg)—empty bone defect/administration of apigenin by gastric gavage in a single dose daily [42,43].

Concentration and solution preparation was performed following a previously described method by Correa et al. [40,41,44]. The resveratrol solution was prepared in 100 mL polysorbate 80 (Sigma-Aldrich, St. Louis, MI, USA), a surfactant and nonionic emulsifier common in pharmaceuticals and food preparation. The curcumin and apigenin solution was obtained in 9% ethanol and diluted in order to obtain the concentrations considered for the present study [40,41,44]. Eventual complications and infections were treated by administering post-operative antibiotic therapy and painkiller therapy. The daily clinical evaluation of the post-operative surgery was performed through the rat grimace scale prior to surgery on Days 1, 3, 7, 14, and 30. The animals were sacrificed after 30 days, and the biopsies were retrieved for further analysis. The obtained samples were radiographically and evaluated by 3D CBCT scans (EZ3D, Vatech, Gyeonggi-do, Republic of Korea) to evaluate the level of bone healing and defect recorticalization.

#### 2.6.2. Specimen Processing

The biopsies were fixed into 4% paraformaldehyde and 0.1% glutaraldehyde in 0.15 M cacodylate buffer and pH 7.4 at room temperature for 1 week. The samples were dehydrated in ascending concentration rinses of ethyl alcohol from 60% to 100% and embedded in a hydrophilic acrylic resin of high viscosity (LR White Resin London Resin Company Ltd., UK). After polymerization, the specimens were sectioned, along their longitudinal axis, with a high-precision and -accuracy diamond disc at about 150 µm and ground down to about 30 µm with a specially designed grinding machine. Two slides were obtained for each specimen. The slides were stained with toluidine blue and acid fuchsin to evaluate the newly formed and mature bone. The samples were observed in normal transmitted light under a Nikon microscope ECLIPSE (Nikon, Tokyo, Japan).

### 2.7. Statistical Analysis

The statistical software package GraphPad 8 (Prism, San Diego, CA, USA) was used for the data analysis. The parametric methods were applied considering the existence of the required assumptions. The study variables were the time elapsed, the molecule concentration, and the gene expression levels. The sample size of the in vivo experiments was calculated for a total of 4 different groups, according to an α error of 0.05 and a power of 80%. The minimum sample size for statistical significance was 5 defects for each group (total of 20 sites and animals). The statistical analysis of the in vitro experiments was conducted by applying the unpaired *t*-Student test. The level of significance was assessed considering a *p* < 0.05. The descriptive statistic of bone defect healing in vivo was calculated by CBCT scans considering the means, standard deviation, and 95% confidence intervals for conditions.

## 3. Results

### 3.1. In Vitro Procedure

#### 3.1.1. Cell Proliferation Assay

**Figure 2:**
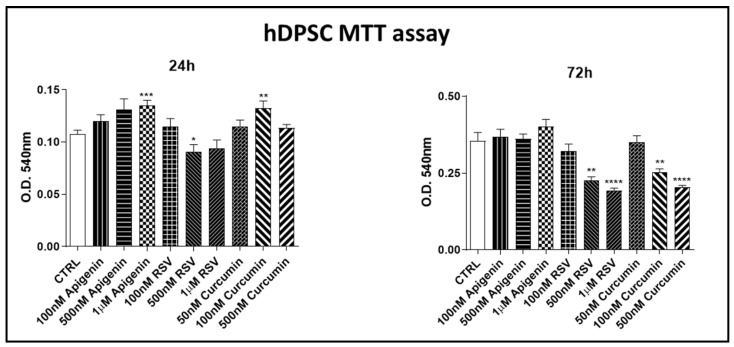
MTT assay on hDPSC cultures at low concentrations. At 24 and 72 h, apigenin was tested at final concentrations of 1 µM, 500 and 100 nM; resveratrol 1 µM, 500 and 100 nM; and curcumin 50, 100, and 500 nM. Data derived from means ± SEM of three different experiments (each *n* = 3), * *p* ≤ 0.05, ** *p* ≤ 0.01, *** *p* ≤ 0.001, **** *p* ≤ 0.0001.

The MTT assay dose–response experiment was assessed to identify the optimal concentration for hDPSC cultures (Figure 2). Apigenin at final concentrations of 1 µM, 500 and 100 nM; resveratrol 1 µM, 500 and 100 nM; and curcumin 50, 100, and 500 nM were tested. The observation was performed at 24 and 72 h to verify the effect after the cells had completed a replication period (Figure 2). To select the most appropriate substance concentrations, we opted for the best concentrations that at both 24 h and 72 h had no toxic effects or with values very similar to controls (CTRL) or even had a proliferative effect. Based on the results obtained, for apigenin, resveratrol, and curcumin, we selected and utilized for further experiments the following concentrations: 1 µM apigenin, 100 nM resveratrol, and 50 nM curcumin.

#### 3.1.2. Alizarin Red Assay

**Figure 3:**
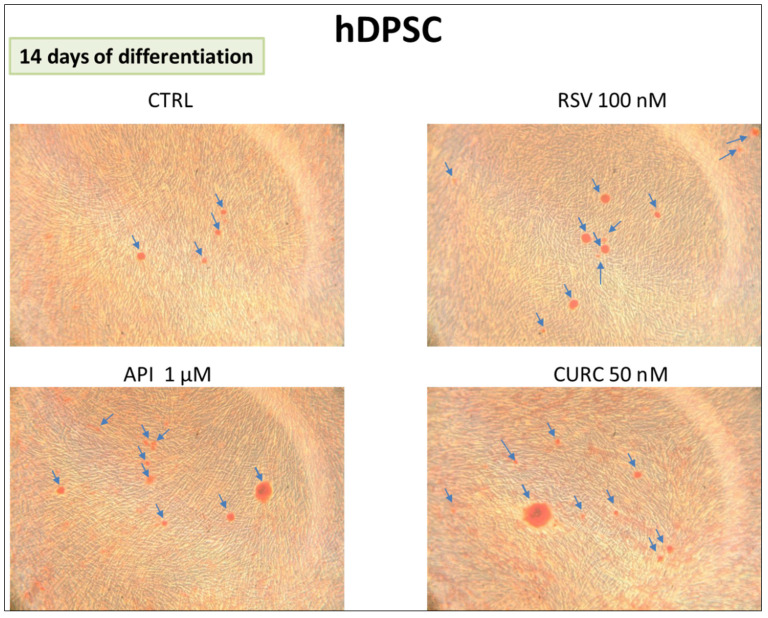
Alizarin Red assay on hDPSC cultures at 14 days of differentiation. The arrows indicate the calcification nodules formation (magnification 10×).

The formation of calcification nodules obtained in hDPSC cultures was shown by Alizarin Red staining. The cells cultured in the presence of 1 µM apigenin or 100 nM resveratrol or 50 nM curcumin were analyzed after 14 days. All samples cultured in the presence of the nutraceuticals showed more nodules positive for Alizarin Red in differentiation medium (DM) conditions with respect to the control (CTRL) (Figure 3).

#### 3.1.3. Gene Expression

**Figure 4:**
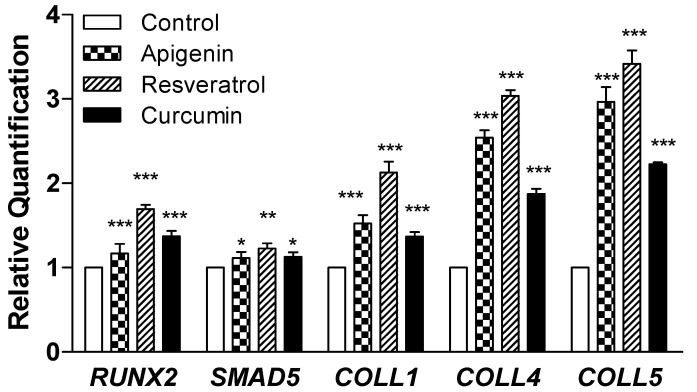
RT-PCR of *RUNX2*, *SMAD5*, *COLL1*, *COLL4*, and *COLL5*. The graph shows the expression of analyzed genes after 14 days of differentiation in DM. Data derived from three different experiments (each *n* = 3) are reported as relative quantification means ± standard error. Unpaired t-test was applied to compare apigenin, resveratrol, curcumin, and the control group (* *p* < 0.05, ** *p* < 0.005. *** *p* < 0.0005).

RT-PCR analysis was performed for *RUNX2*, *SMAD5*, *COLL1*, *COLL4*, and *COLL5* genes after 14 days of differentiation in DM, and the data were reported as relative quantification means ± standard error (Figure 4). The expression of genes *SMAD5* and *RUNX2* was statistically increased in the presence of apigenin, resveratrol, and curcumin compared to the control group (*p* < 0.05). Even *COLL1*, *COLL4*, and *COLL5* were upregulated in the presence of apigenin, resveratrol, and curcumin compared to the control (*p* < 0.05).

#### 3.1.4. In Vivo Procedure

No dropouts were reported after the surgery and the treatment protocol. A good tolerance of the treatment was evidenced for all time points on Days 1, 3, 7, 14, and 30, with no difference between the treatment groups (rat grimace scale) (*p* < 0.05) (Table 1). No evidence of infection or inflammation was present in all groups.

#### 3.1.5. Tomography Assessment 

**Figure 5:**
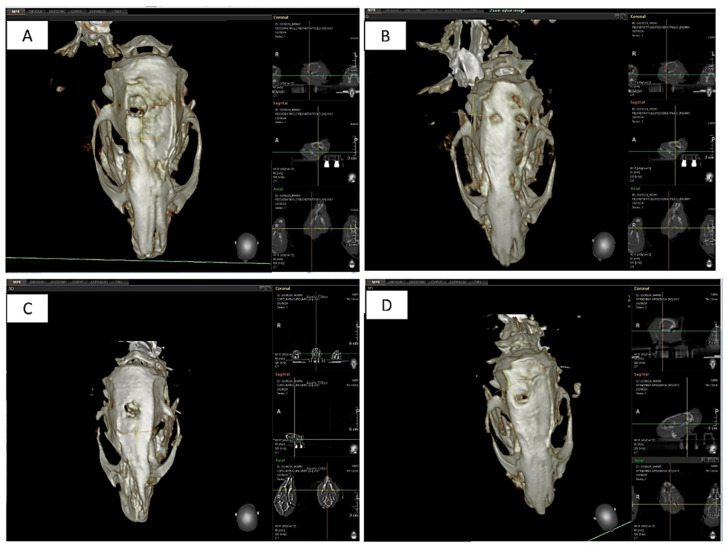
Details of the cone beam tomography scans of the specimens after 30 days of treatment. (**A**) Control no treatment after 30 days; (**B**) apigenin treatment with daily administration after 30 days; (**C**) curcumin treatment with daily administration after 30 days; (**D**) resveratrol treatment with daily administration after 30 days.

All defects showed a bone repair at 30 days from the surgery visible by CBCT scans and optical microscopy. After 30 days of treatment, a significantly improved corticalization was visible for apigenin (Figure 5B) compared to the resveratrol (Figure 5C) and curcumin groups (Figure 5D) after the healing period, while this evidence was not evident in the control group (Figure 5A) (*p* < 0.05). No evidence of fibrous tissue was present in all specimens at the microscopic observation.

#### 3.1.6. Histological Assay

**Figure 6:**
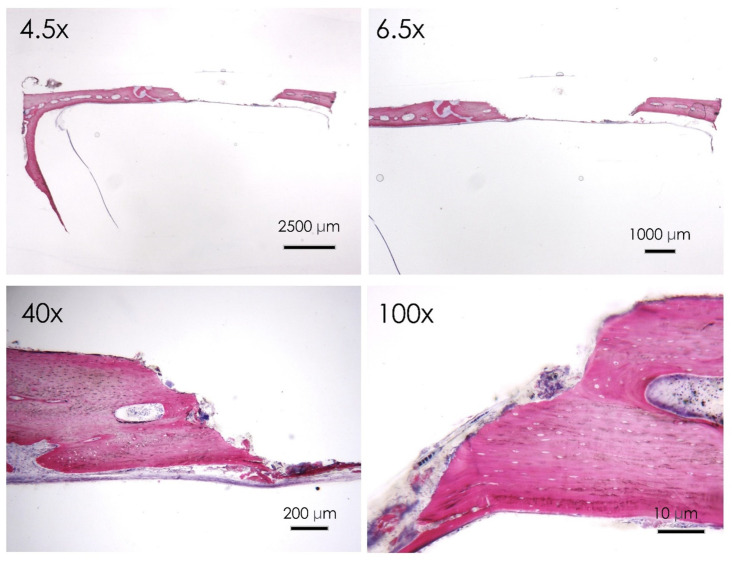
Control group. Histological section of the calvaria bone specimens at different magnifications (4.5×, 6.5×, 40×, and 100×). Evidence of surface bone resorption was evident at the level of the defect margin.

All the samples treated healed normally with no evidence of infection or inflammatory infiltrate. The histological evaluation with acid fuchsin and toluidine blue staining showed that after 30 days from the surgery, the control group reported evidence of marginal bone resorption with a few areas of new bone formation localized in the intracranial side of the cortical bone. Active multinucleated osteoclasts activity is evident at the level of the marginal walls of the defect. No evidence of inflammatory infiltrate was reported at the higher magnification (Figure 6) level of the margin of the defect. Focal regions of new bone formation were present at the level of the intracranial side of the defect.

**Figure 7:**
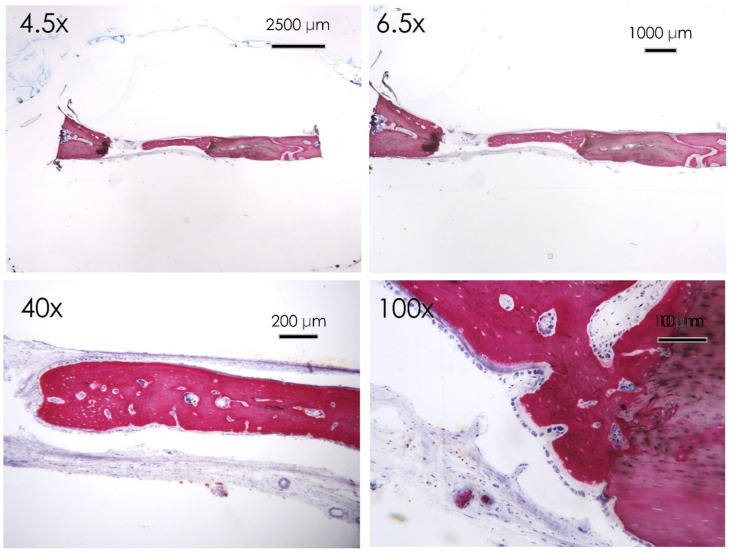
Apigenin group. Histological section of the calvaria bone specimens at different magnifications (4.5×, 6.5×, 40×, and 100×). A consistent filling of the bone defect was evident after 30 days of apigenin administration.

**Figure 8:**
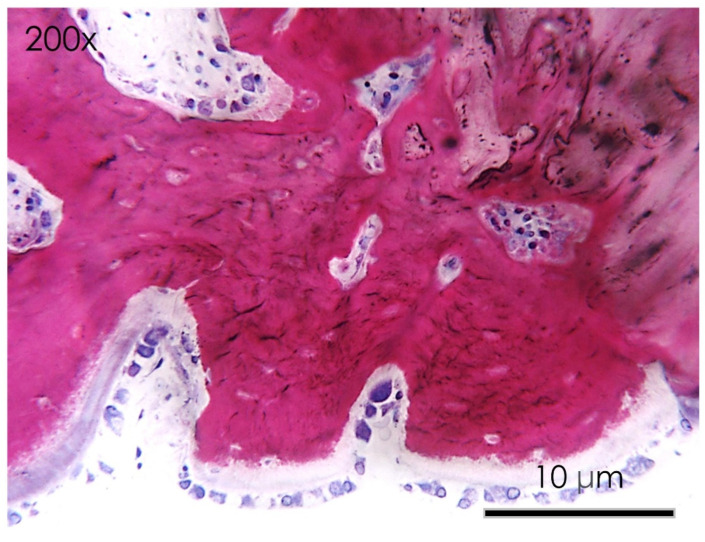
APIGENIN GROUP. Detail at higher magnification of the histological section of the calvaria bone specimens (200×). An active process of bone remodeling of the osteoid matrix was evident.

In the apigenin group, bone morphology presented differentiated cell lineages specific to the bone tissues, such as osteoblasts, osteocytes, and newly formed blood vessels. The histological images showed a wide number of newly formed bone trabeculae (Figure 6 and Figure 7), while osteoblasts actively secreted the osteoid matrix that, in some areas, was undergoing mineralization. At higher magnifications, an osteoclast rim was present at the level of the defect margins with evidence of an active remodeling of the bone tissue (Figure 8).

**Figure 9:**
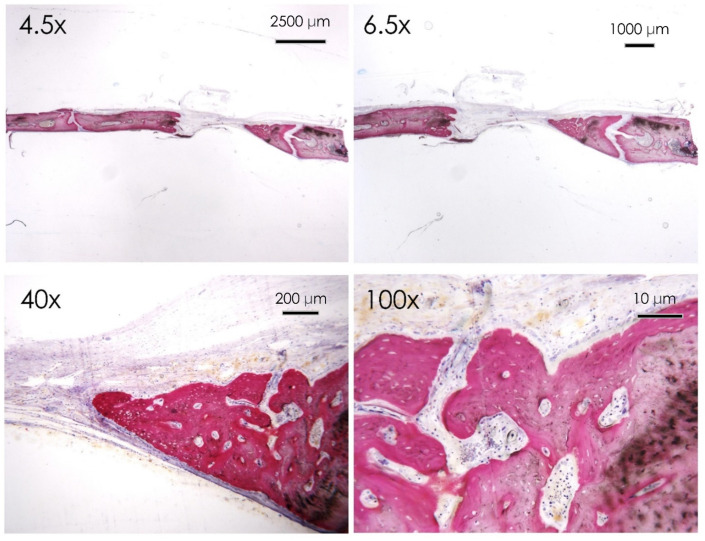
Resveratrol group. Histological section of the calvaria bone specimens at different magnifications (4.5×, 6.5×, 40×, and 100×). A filling of the bone defect was reported after 30 days of resveratrol administration.

**Figure 10:**
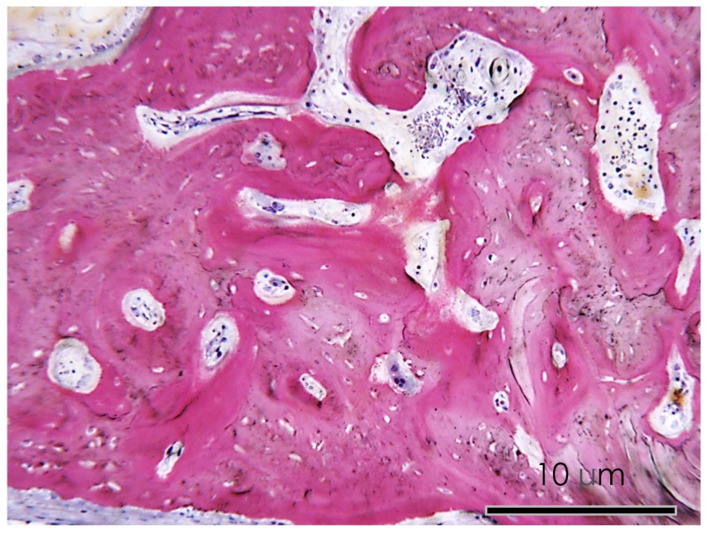
Resveratrol group. Detail at higher magnification of the histological section of the calvaria bone specimens (200×). A quantity of bone lacunae and osteoid matrix apposition was reported.

In the resveratrol group no fibrous tissue was observed at the level of the newly formed bone surfaces compared to the control group (Figure 9 and Figure 10). Newly formed bone was found in close contact with the calvarial defect margins with wide bone trabeculae and large osteocyte lacunae (Figure 10). The osteoblasts were actively secreting the osteoid matrix that, in some areas, was undergoing mineralization.

**Figure 11:**
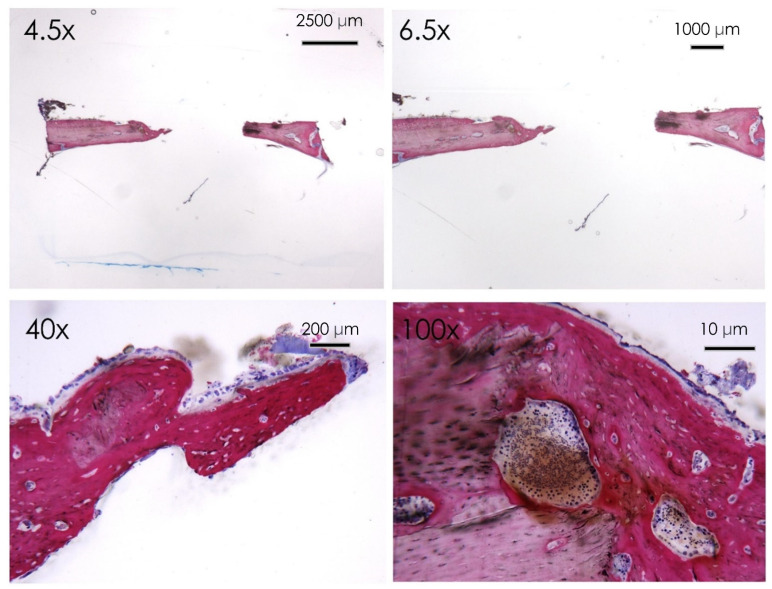
Curcumin group. Histological section of the calvaria bone specimens at different magnifications (4.5×, 6.5×, 40×, and 100×). A noncomplete filling of the bone defect was evident after 30 days of curcumin administration.

**Figure 12:**
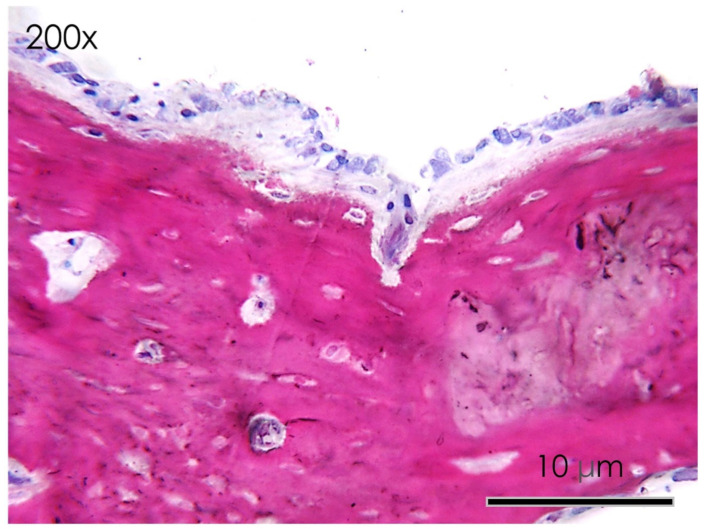
Curcumin group. Detail at higher magnification of the histological section of the calvaria bone specimens (200×). A nonorganized osteoid matrix neo-apposition was reported.

In the curcumin group, a noncomplete filling of the bone defect was evident after 30 days of curcumin administration, and a nonorganized osteoid matrix neo-apposition was reported (Figure 11 and Figure 12).

#### 3.1.7. Bone Defect Repair

**Figure 13:**
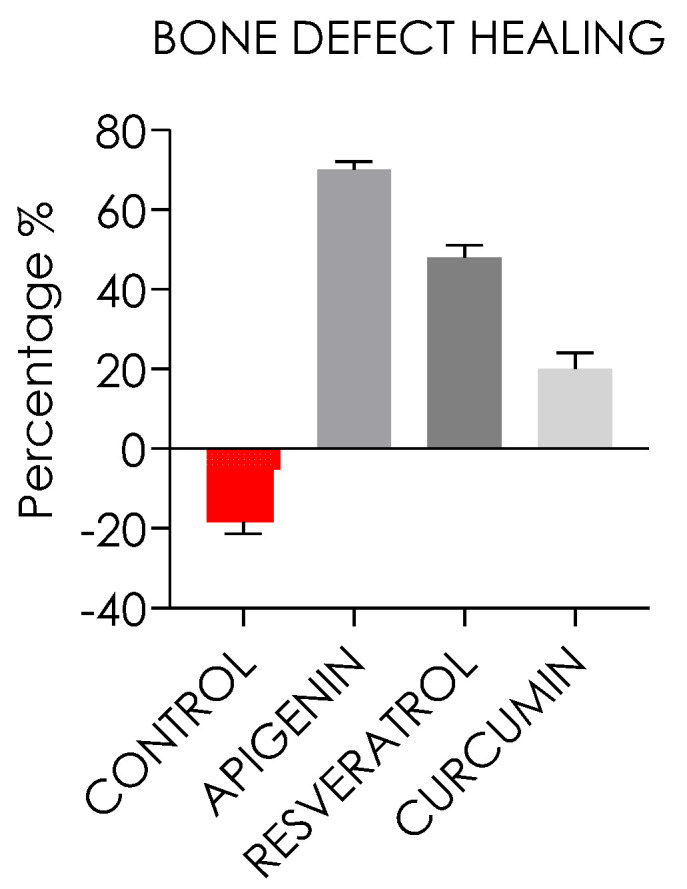
Bone defect healing of the control, apigenin, resveratrol, and curcumin groups after 30 days from the surgery. A negative defect repair of the control group was present, while the apigenin administration showed the highest level of defect healing.

The bone defect radiographically measured reported that the control group showed a significantly lower bone repair level compared to the experimental groups with a mean resorption of 18.5 ± 2.4% (*p* < 0.05). The bone defect repair of the apigenin group was 69.6 ± 2%. The bone defect healing of the resveratrol group and curcumin groups was, respectively, 42.5 ± 3.6% and 23.7 ± 4.1% smaller than the baseline diameter defect, as reported in Figure 13.

The defect area treated with apigenin gave the highest new bone formation with respect to the other treated groups. In the apigenin, resveratrol, and curcumin groups, a higher new bone formation was detected compared to the control group. The curcumin group showed a lower percentage of bone compared to apigenin and resveratrol after 30 days of treatment, but even higher than the control group (Figure 13).

## 4. Discussion

The functional and aesthetic restoration of bone defects and atrophies represents a clinical condition that could require a regenerative approach and grafting procedures [3,45,46]. The identification of novel approaches and adjuvant therapies able to improve the healing of damaged tissues and bone defects represents one of the recent orientations of regenerative medicine [47,48,49]. Several studies have been performed to verify the regenerative effects of some plant-derived substances, such as phenolic compounds, both in vitro and in vivo in different experimental models [26,50,51]. However, no studies have been carried out that simultaneously analyze these three compounds in parallel and which pathways downstream of *RUNX2* are modulated in the process of osteogenesis. Furthermore, even in vivo, there is no research investigating the parallel use of these natural compounds. The only study involving these three phenolic compounds concerns the effects of these nutraceuticals in inducing cancer signaling pathway manipulation and possibly facilitating new treatment modalities for osteosarcoma [39].

The present compound concentrations were adopted considering the evidence of the literature in this field. The dosages used demonstrated their effectiveness in very wide experimental models on rats, but the critical-size bone defect represents a novelty of the present paper. Correa et al. investigated the same concentration of nutraceutical administered by daily gastric gavage to evaluate in an experimental model of periodontitis, validating their effectiveness and safety with no adverse effect on rats [40].

In addition, apigenin and curcumin administered orally demonstrated in two different studies a consistent effectiveness for bone loss prevention and mineral density on ovariectomy-induced bone loss in rats [41,43]. Low-dose daily administration is a strategy that is able to avoid the risk of high-dosage compound administration that could produce significant systemic side effects. On the contrary, gastric absorption could represent a potential weak point due to the compound absorption levels and the peripheric balance levels necessary to produce a therapeutic level [40,41,43].

Among the most studied bioactive compounds is certainly resveratrol, as well as other molecules such as apigenin, a natural flavone, and curcumin with beneficial effects on cell differentiation [38,52]. A fairly recent study reports that apigenin, at µM concentration, stimulates myogenic differentiation of the murine cell line C2C12 and regulates the expression of total myosin heavy chain (*MHC*), *MHC2A*, and *MHC2B* [53]. In the present investigation, we evaluated the putative role of apigenin, resveratrol, and curcumin dietary supplements in improving the in vitro osteogenic differentiation of hDPSCs, as well as in affecting bone healing with an improvement of the clinical outcomes in vivo. The in vitro results of the study showed that apigenin, resveratrol, and curcumin could provide a promoting action for osteogenetic proliferation and differentiation of hDPSCs. The positivity for Alizarin Red staining showed mineral deposition obtained from hDPSCs cultured either in the presence or not of apigenin, resveratrol, and curcumin. The calcification nodules were more observable after 14 days of culture in DM in the presence of stimuli, demonstrating an earlier differentiation of hDPSCs in an osteogenic pattern. These results were in line with previous outcomes showing positive alizarin-red-stained calcification nodules in 21-day-differentiated hDPSCs [54]. However, the more pronounced calcification nodules In the presence of stimuli confirmed an earlier differentiation process related to their effect. We can speculate that the three substances were effective in boosting osteogenic differentiation both in in vitro and in vivo experiments. Indeed, we demonstrated that the hDPSCs cultured in the presence of the nutraceuticals showed more nodules positive for Alizarin Red in differentiation medium conditions with respect to the control. Moreover, in vivo, in the apigenin, resveratrol, and curcumin groups, a higher new bone formation was detected compared to the control group.

Multipotent stem cells are induced to osteogenic differentiation into osteoblasts according to *BMP* signaling [55,56,57]. *BMP2* signaling appears to permit the expression of osteo-specific *RUNX2*, which induces the expression of alkaline phosphatase and osteocalcin, and *SMAD5*, which activates the expression of early osteoblast differentiation markers [57,58,59]. Moreover, *BMP2* and *SMAD5* can bind *RUNX2* with an increase in transcriptional activity [57]. In fact, *RUNX2* activity with osteocalcin and β-catenin is known to be essential for osteoblast formation [60,61,62]. The activation of this signaling is confirmed by the increased upregulation of *SMAD5* and *RUNX2*. The study data confirmed the literature showing the maximum upregulation at 14 days of culture [7]. *RUNX2* is a determinant transcription factor in bone formation and upregulates osteocalcin, a key regulator of the development of the osteoblast phenotype by modulating bone extracellular matrix proteins and collagen types 1, 4, and 5. Indeed, its increased activity could suggest a more rapid evolution and a major degree of differentiation of hDPSCs on the osteogenic line [60,61,62]. In agreement with the study findings, resveratrol oral administration is able to decrease alveolar bone resorption in experimental defects with reduced levels of proinflammatory cytokines *IL1b*, *IL6*, and *TNFa* [33,43]. In addition, in the literature, treatment with curcumin in experimental bone defects has been shown by others to downregulate *RANKL/RANK/osteoprotegerin*, as well as to reduce bone loss [33]. These physiopathogenetic mechanisms seem to be correlated with the regulation of various molecular targets, including increasing ALP activity and osteoblast-specific mRNA expression of *RUNX2* and osteocalcin [29,63,64]. Curcumin is able to decrease the release of inflammatory cytokines in articular chondrocytes and produce antagonist activity against proinflammatory molecules [33]. As reported in the literature, apigenin, curcumin, and resveratrol have potent antioxidant activity [20,30,33,43,49,65]. The cell cultures exhibit upregulation of all tested genes in the presence of nutraceutical administration during osteogenic differentiation, which may be associated with augmented cell activity and growth. In order to understand if the enhanced differentiative features demonstrated in vitro on hDPSC cultures could be related to the more effective healing of bone defects, we also evaluated in vivo in rats the effects of apigenin, resveratrol, and curcumin administration on experimental critical-size bone defects. Park et al. reported an ovariectomy-induced bone loss in rats, where the administration of apigenin with a dose of 10 mg/kg three times a week for 15 weeks induced an increase in the bone density of the trabecular bone of the rat femur, with an inhibition of bone resorption and osteoclast apoptosis [43,66]. In agreement with the current literature, we used 10 mg/kg of apigenin, curcumin, and resveratrol. Our data showed that apigenin seems to be more effective at enhancing the healing of bone defects and mineralization of the osteoid matrix in rat calvaria compared to the control group. Moreover, the apigenin group resulted in more effective and significantly higher bone defect repair compared to the other study groups. The control group revealed after 30 days a marginal bone resorption that is compatible with a local adaptation that produced a significant increase in the bone defect diameter. Conversely, mild new bone formation activity was localized in the intracranial portion of the defect.

Another notable aspect is the histological findings of the initial active remodeling process of the osteoid matrix visible in all test groups compared to the control, which reported evidence of marginal bone resorption activity. These results are in agreement with previous studies reported in the literature [26,33,43]. In addition, the findings of the in vivo experiment confirmed the rejection of the null hypothesis, revealing a significant difference concerning the percentage of calvarial defect repair and the histological evidence of a more active nonmature bone synthesis of the test groups.

## 5. Conclusions

Apigenin, curcumin, and resveratrol investigated in parallel in the present study showed a significant increase in bone repair in critical-size defects in rat calvaria. Among these, apigenin induced the best results. These in vivo results could be related to a differentiative boost due to all substances, as shown by the in vitro results. Altogether, the study evidence encourages a translational application for fracture defect repair as adjuvant supplement therapy. It is noteworthy that in the present study, gastric gavage was used as a new method for substances’ administration in in vivo experiments, intended to mimic the effects that could be obtained with dietary supplementation of possible therapeutic integrations with nutraceuticals during the bone regeneration process. Based on the findings of the present investigation, further longer-term studies in models of experimental bone defects might be required to elucidate the effects of nutraceuticals. This could lead to the development of more effective therapies and adjuvant supplements approaches for bone defect repair in humans.

## Figures and Tables

**Table 1:** Summary of the rat grimace scale at 1, 3, 7, 14. and 30 days for the control, apigenin, resveratrol, and curcumin groups.

Rat Grimace Scale (Mean, SD)	Day 1	Day 3	Day 7	Day 14	Day 30
Control Group	1.7 ± 0.4	1.1 ± 0.5	0.6 ± 0.4	0.3 ± 0.4	0.2 ± 0.4
Resveratrol Group	1.7 ± 0.5	1.2 ± 0.5	0.6 ± 0.5	0.2 ± 0.4	0.2 ± 0.4
Apigenin Group	1.8 ± 0.4	1.1 ± 0.6	0.6 ± 0.4	0.3 ± 0.5	0.2 ± 0.5
Curcumin Group	1.7 ± 0.4	1.2 ± 0.5	0.6 ± 0.4	0.2 ± 0.5	0.2 ± 0.4
*p* value	*p* > 0.05	*p* > 0.05	*p* > 0.05	*p* > 0.05	*p* > 0.05

## Data Availability

All experimental data to support the findings of this study are available contacting the corresponding author upon request. The authors have annotated the entire data building process and empirical techniques presented in the paper.
